# Supraclavicular Cervical Schwannoma: A Case Report

**DOI:** 10.7759/cureus.5924

**Published:** 2019-10-16

**Authors:** Mohammed Elkarim Mohammed, Abdullatif Khan, Mohammed Asiri

**Affiliations:** 1 Otolaryngology, King Abdulaziz Medical City, National Guard Health Affairs, Riyadh, SAU; 2 Pathology, King Abdulaziz Medical City, National Guard Health Affairs, Riyadh, SAU

**Keywords:** schwannoma, supraclavicular, cervical schwannoma, neck mass

## Abstract

Schwannoma generally occurs in the vestibular nerve. Cervical schwannomas are quite rare. Herein, we present a case of slow-growing asymptomatic supraclavicular schwannoma in an elderly woman of 74 years. The patient was managed conservatively with regular follow-up as the tumor showed slow progressive growth (presented after seven years) and asymptomatic.

## Introduction

Schwannoma, also known as neurilemmoma, is a very slow-growing benign tumor arising from the Schwann cells [1-3]. The Schwann cells (discovered by and named after German physiologist Theodor Schwann) or the neurolemmocytes are the most important glial cells of the peripheral nervous system as they provide insulation and help salutatory conduction of action potential through axons [1-2]. Schwann cells are located anywhere along the entire course of a nerve and can arise from any of the cranial nerves (from third to twelfth cranial nerves) or autonomic or peripheral nerves. The most common type of Schwannoma arises from the vestibular nerve presenting with unilateral hearing loss on the affected side and tinnitus [1-4]. Although not so common, cervical schwannoma can be incidentally discovered on postmortem in 3% to 4% of the patients [1-4].

Schwannomas arising from the brachial plexus are quite rare; only 5% of all the Schwannoma cases arise from the brachial plexus [5]. The location of schwannoma at this site poses diagnostic challenges as it is often confused with cervical lymphadenopathy.

In this case report, we describe a case of cervical Schwannoma and also present a review of the existing literature in this regard.

## Case presentation

Herein, we present a case of cervical schwannoma in a 74-year-old elderly female. She reported with a longstanding swelling in the left collar bone area (supraclavicular region). The swelling persisted for more than seven years. She did not complain of weakness, abnormal sensation, or numbness in the left hand.

On clinical examination, it was found that the swelling was firm in nature, non-tender, and non-pulsatile. It was located at the level 5 region and measured 4 ×4 cm. No other neck masses, such as cervical lymph nodes, were palpable.

Neck computed tomography (CT), magnetic resonance imaging (MRI) with contrast, and fine-needle aspiration cytology (FNAC) were performed.

CT and MRI findings

**Figure 1:**
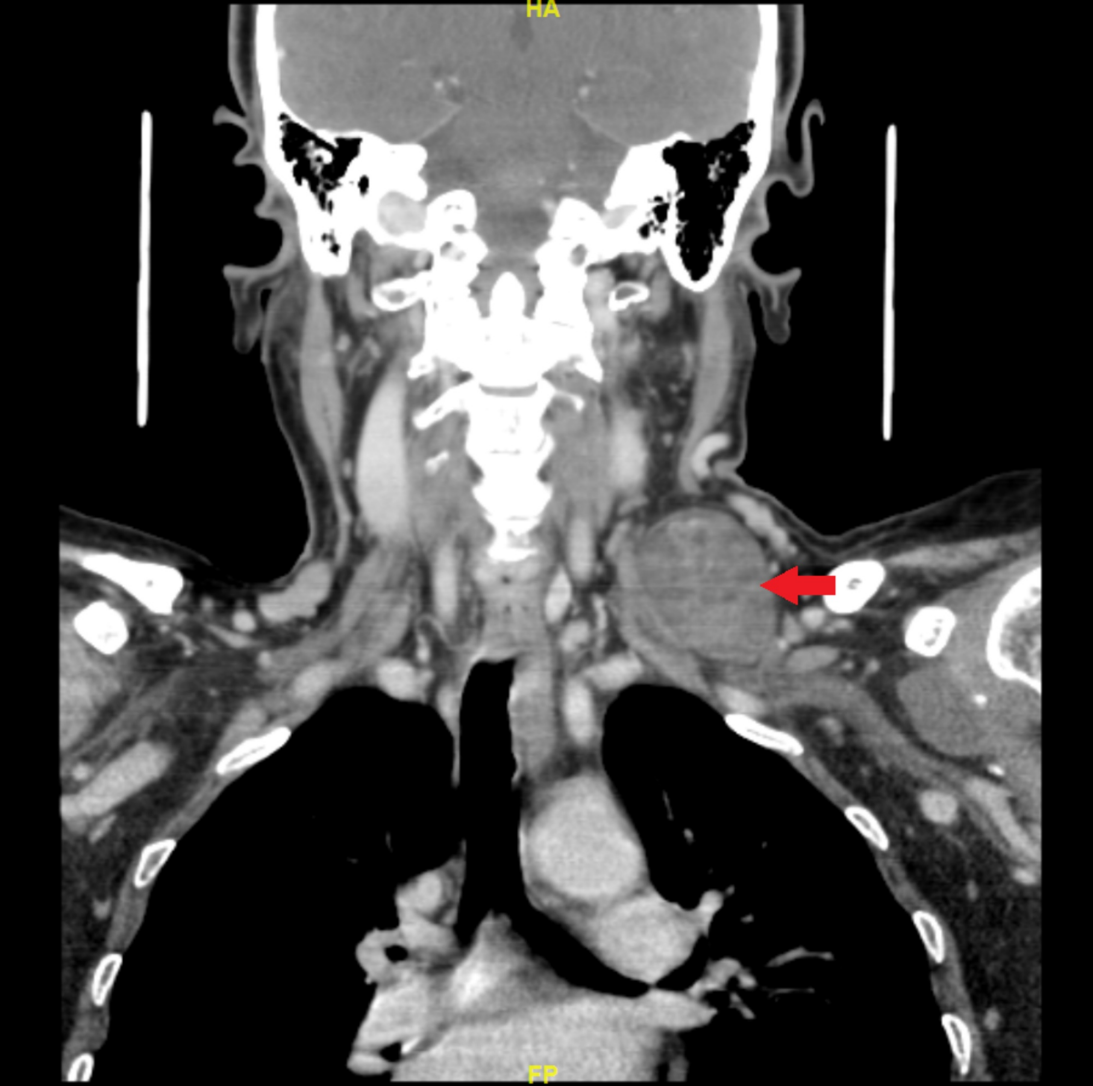
Contrasted coronal CT of the neck A left-sided supraclavicular well-circumscribed mass measuring 3.5 × 4 cm (arrow) CT, computed tomography

CT scan revealed a large well-defined mass located in the left supraclavicular region. It measured 3.5 ×3.5 cm (transverse × anteroposterior measurements; Figure 1).

**Figure 2:**
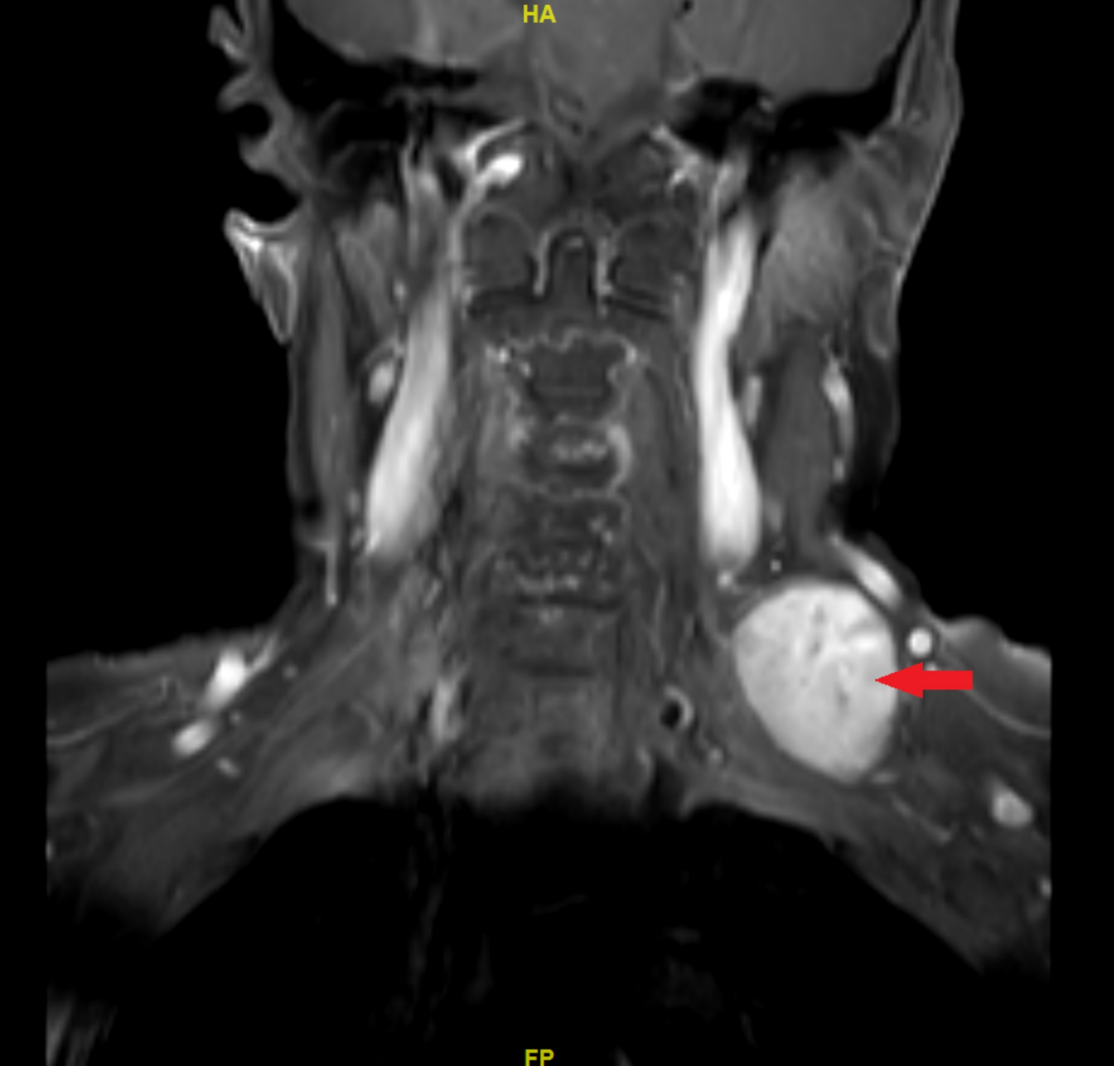
Contrasted coronal T1 MRI of the neck A left-sided well-circumscribed hyperintense neck mass (arrow) MRI, magnetic resonance imaging

**Figure 3:**
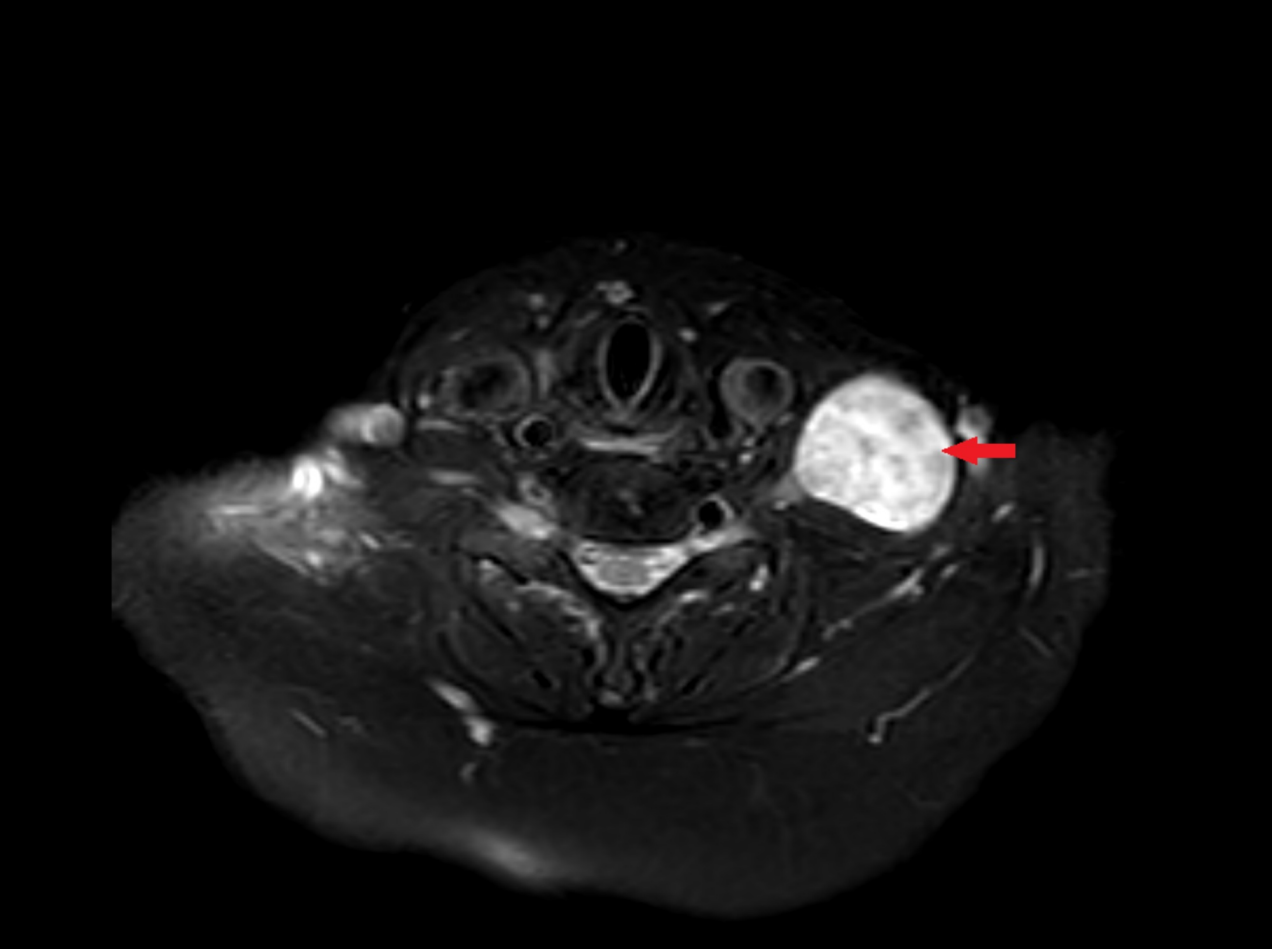
Axial T2 MRI of the neck A left-sided, well-circumscribed hyperintense neck mass (arrow) MRI, magnetic resonance imaging

Further, MRI scan revealed a low T1 signal with a high T2 signal with heterogeneous enhancement (Figures 2-3). The tumor was also found to be in continuity with the peripheral nerves located at the C5 and C6 levels in close proximity with the upper part of the left brachial plexus. No other neck masses were found and lymphadenopathy was also absent.

Both MRI and CT scan findings were consistent with the diagnosis of Schwannoma.

FNAC findings

**Figure 4:**
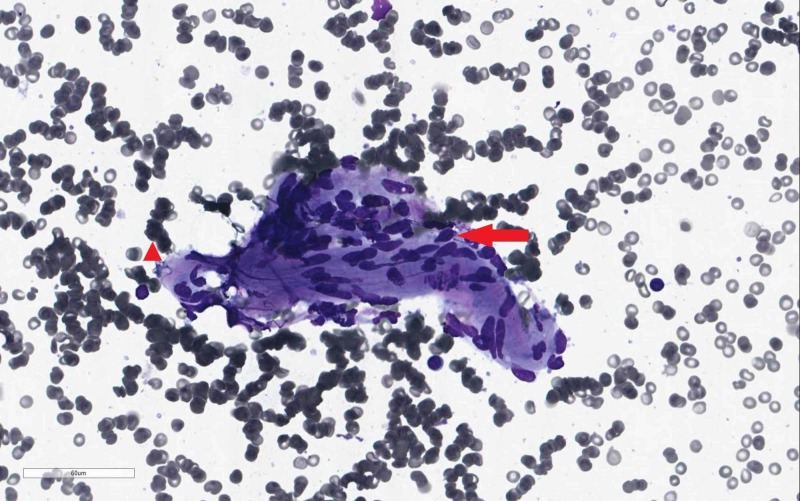
FNAC result Clusters of cohesive epithelioid histocytes (arrow) in the background of lymphocytes (arrow head) FNAC, fine-needle aspiration cytology

FNAC of the lesion revealed clusters of cohesive epithelioid histiocytes in the background of lymphocytes with no malignant cells suggestive of a benign spindle cell neoplasm (Figure 4).

As the patient was asymptomatic, conservative management was advised.

## Discussion

This was a purely observational study; hence, patient management was done through regular follow-up only. Therefore, ethical permission was not taken. However, written consent from the patient was obtained for publication of the case report along with the images.

Neurogenic tumors arise from the neural crest cells. However, Schwann cells differentiate from neural crest cells along with sympathicoblasts [1-3]. Two types of tumors arise from the Schwann cells: neurofibroma and Schwannoma [1-3].

Schwannoma or neurilemmoma are benign, slow-growing tumors. These tumors are typically encapsulated and remain attached to the nerves from its origin. Schwannoma typically emerges from any type of nerve (cranial and spinal) with a sheath except for optic and olfactory nerves, which lack a myelin sheath. Nearly 45% of all schwannomas occur in the head and neck area [6].

In 1908, Jose Verocay (“the Prague Pathologist”) first established Schwannoma as a pathological condition [7]. He first described the pathological structures known as the Verocay body’s, a histopathological diagnostic feature of Schwannoma and named it neurinoma. The term neurilemmoma was first used by Stout in the year 1935 [7].

In the head and neck region, around 25 to 40% of all the Schwannomas appear in the parapharyngeal space [8]. In the year 1933, the first case of Schwannomas in the parapharyngeal space was reported by Figi [9]. Although quite rare, the occurrence of Schwannomas in the head and neck region also includes the submandibular area, paranasal sinuses, cheek, and oral cavity [6].

The tumors might vary in size ranging from a few mm in size to 24 cm. Signs and symptoms presentation may also vary depending upon the anatomical location of the tumors. Most of the patients present with painless swelling. However, if present, symptoms usually depend upon the site of the tumor. Other than pain, a tingling sensation and numbness along the course of the involved nerve are also reported. Other symptoms could be breathing difficulty, especially, when the tumor is located in the nose, painful swallowing in case a tumor in the pharynx, nose bleeding due to the presence of a tumor in paranasal sinuses, breaking of voice when a tumor is located in the larynx, or just a swelling in the neck in case of a tumor in the parapharyngeal space [10]. In most of the cases, the swelling is freely mobile. However, attachment to nerves might lead to limited mobility of the tumor. In some cases, the nerves from which the tumor has originated could be completely encompassed in the Schwannomas [6].

Schwannoma can affect people of any gender and age. However, the most common age of occurrence is in the third and fourth decades of life [10]. In the neck region, Schwannomas has been categorized depending upon the groups of nerves involved; these are medial and lateral groups [1]. Medial groups of Schwannomas arise from either the cervical sympathetic chain or from any of the last four cranial nerves. On the contrary, the lateral groups of schwannomas arise from the trunk of the cervical nerve, cervical plexus or from the brachial plexus [11]. These tumors might also arise from vagus nerve or glossopharyngeal nerve.

In the present case, a 74-year-old woman presented with a painless swelling in the left supraclavicular region. The swelling gradually appeared over a period of seven long years. The tumor location assessment allowed categorizing the tumor in the lateral group of tumors and also suggested that the tumor probably arising from cervical or brachial plexus. Radiological findings (MRI) revealed that the tumor had emerged from peripheral nerves in close proximity to the upper part of the brachial plexus.

Brachial plexus tumors are rare in occurrence; less than 5% of the upper extremity Schwannomas originate from brachial plexus [4]. In 1995, Kehoe and colleagues reported that around 36% of the Schwannomas emerging from the brachial plexus present as supraclavicular mass and rest present as infraclavicular mass [11].

Confirmation of the diagnosis of Schwannomas located in the head and neck region before surgery is rather difficult. Commonly performed investigations include CT, MRI, and FNAC. MRI is an admirable diagnostic tool with a number of advantages including better soft-tissue contrast and excellent three-dimensional modeling. CT scan is useful if the tumor originates from bony structures like pterygoid plates or posterior body plates of the maxillary sinuses [12].

If the situation allows, diagnosis can be confirmed by FNAC. Common diagnostic features include the presence of fibrous capsule, Antoni A and Antoni B areas, and Verocay bodies. In larger and older Schwannomas, degenerative changes, vascular sclerosis, and hemorrhagic changes are common. Sometimes, the formation of a microcyst is evident with a pseudoepithelial lining of the Schwann cells.

In 2015, Yafit et al. published an algorithm regarding the choice of treatment for extracranial Schwannomas located in the head and neck region [13]. According to the algorithm, treatment options include only observation and regular follow-up in asymptomatic patients, surgical intervention in symptomatic cases, and in patients unfit for surgery, palliative radiotherapy could be opted [13].

In our case, as the patient was asymptomatic, a conservative approach was adopted. Similar to our case, Keleş E and his colleagues presented a case report of the supraclavicular area, wherein a 71-year-old man presented with large swelling in the left supraclavicular area [1]. Although the tumor was painless and the patient did not complain of any other neurological symptoms, the mass was excised, unlike our case which was also painless and asymptomatic. The reason behind the surgical intervention in their patient is probably due to the fact that the tumor size was 6 × 4 cm over a period of only six months. By contrast, our patient reported that the swelling was there for a prolonged period of seven years and the size was comparatively less (4 ×4 cm).

Similar to our case, Ando et al. also presented a retrospective study with 23 Schwannoma cases diagnosed by MRI and concluded that majority of the asymptomatic Schwannomas are very slowly progressing and do not require surgical intervention, rather should be closely monitored for any increase in size or appearance of nerve compression [14].

Common differential diagnoses include a tumor of the carotid body, cervical lymphadenopathy, thyroid cyst or nodule, teratoma, brachial cysts, lipoma, metastatic deposits, neurofibroma, etc. Location of the tumor, CT, MRI, and FNAC help in the establishment of diagnosis [1,15].

## Conclusions

In elderly patients, presenting with supraclavicular swelling, thorough clinical examination and investigations should be carried out to rule out other conditions especially malignancy. Asymptomatic slow-growing Schwannomas can be managed conservatively with careful observations and regular follow-ups.

## References

[1] Keleş E, Eroğlu O, Özercan İH, Özel İ (2018). Schwannoma in the supraclavicular region: case report. Turk Arch Otorhinolaryngol.

[2] Vučemilo L, Lajtman Z, Mihalj J, Plašćak J, MahovićLakušić D, Mužinić D (2018). Brachial plexus schwannoma - case report and literature review. ActaClin Croat.

[3] Terada Y, Toda H, Yokote A, Iwasaki K (2016). A mobile schwannoma of the cervical spinal cord: case report and review of the literature. Neurosurgery.

[4] Ryu KH, Moon JI, Baek HJ, Cho SB, Choi BH, An HJ, Song DH (2018). Brachial plexus schwannoma mimicking cervical lymphadenopathy: a case report with emphasis on imaging features. Medicine.

[5] Kumar A, Akhtar S (2011). Schwannoma of brachial plexus. Indian J Surg.

[6] Das J, Saha J, Dutta S, Manickam A (2016). Cervical schwannoma-a case report. Otolaryngol.

[7] Bologna-Molina R, Vigil-Bastitta G, Pereira-Prado V, Elola-Verocay L (2018). JOSE VEROCAY - "Pragues pathologist". The history of a Latin-American doctor. CeskPatol.

[8] Weber AL, Montandon C, Robson CD (2000). Neurogenic tumors of the neck. RadiolClin North Am.

[9] Neville BW, Damm DD, Allen CM, Bouqot JE (2008). Oral and Maxillofacial Pathology 3rd Edition. Oral and maxillofacial pathology. 2nd edn.

[10] Valentino J, Boggess MA, Ellis JL, Hester TO, Jones RO (1998). Expected neurologic outcomes for surgical treatment of cervical neurilemomas. Laryngoscope.

[11] Kehoe NJ, Reid RP, Semple JC (1995). Solitary benign peripheral-nerve tumours. Review of 32 years' experience. J Bone Joint Surg Br.

[12] Callum Farris (2011). Review of Scott Brown’s Otorhinolaryngology, Head and Neck Surgery, 7th edn. Ann R Coll Surg Engl.

[13] Yafit D, Horowitz G, Vital I, Locketz G, Fliss DM (2015). An algorithm for treating extracranial head and neck schwannomas. Eur Arch Otorhinolaryngol.

[14] Ando K, Imagama S, Ito Z (2016). How do spinal schwannomas progress? The natural progression of spinal schwannomas on MRI. J Neurosurg Spine.

[15] Biswas D, Marnane CN, Mal R, Baldwin D (2007). Extracranial head and neck schwannomas--a 10-year review. Auris Nasus Larynx.

